# Single-Domain Antibody-Based TCR-Like CAR-T: A Potential Cancer Therapy

**DOI:** 10.1155/2020/2454907

**Published:** 2020-09-07

**Authors:** Lichen Zhu, Xiaomei Yang, Dani Zhong, Shenxia Xie, Wei Shi, Yangzi Li, Xiaoqiong Hou, HuaYao, Huihui Zhou, Minlong Zhao, Ziqiang Ding, Xinyue Zhao, Fengzhen Mo, Shihua Yin, Aiqun Liu, Xiaoling Lu

**Affiliations:** ^1^Nanobody Research Center, Guangxi Medical University, Nanning, Guangxi 530021, China; ^2^School of Preclinical Medicine, Guangxi Medical University, Nanning, Guangxi 530021, China; ^3^Department of Chemotherapy, Affiliated Cancer Hospital, Guangxi Medical University, Nanning, Guangxi 530021, China; ^4^Guangxi Collaborative Innovation Center for Biomedicine, Guangxi Medical University, Nanning, Guangxi 530021, China; ^5^School of Stomatology, Guangxi Medical University, Nanning, Guangxi 530021, China

## Abstract

Retargeting the antigen-binding specificity of T cells to intracellular antigens that are degraded and presented on the tumor surface by engineering chimeric antigen receptor (CAR), also named TCR-like antibody CAR-T, remains limited. With the exception of the commercialized CD19 CAR-T for hematological malignancies and other CAR-T therapies aiming mostly at extracellular antigens achieving great success, the rareness and scarcity of TCR-like CAR-T therapies might be due to their current status and limitations. This review provides the probable optimized initiatives for improving TCR-like CAR-T reprogramming and discusses single-domain antibodies administered as an alternative to conventional scFvs and secreted by CAR-T cells, which might be of great value to the development of CAR-T immunotherapies for intracellular antigens.

## 1. Introduction

Cancer diseases, setting up barriers to human longevity worldwide, are estimated to be the top cause of death, based on the most recent GLOBOCAN data [1]. Scientists are working to control and even eliminate cancer through activating and enhancing immunity. From the initial unsuccessful trial of “Coley toxin” immunotherapy in 1893 to the top scientific breakthrough published in *Science* in 2013, a new era of immunooncology has arrived. Immunooncology has even been nominated for the first prize in 2016's MIT top 10 Breakthrough Technologies [2–4]. With the increasing number of tumor antigens and autoimmune T cell receptors gradually being discovered and identified, associated immune checkpoint monoclonal antibodies including Ipilimumab (anti-CTLA-4 mAb) and Pembrolizumab and Nivolumab (anti-PD-1 mAb) have been approved by the Food and Drug Administration (FDA) [5]. Moreover, personalized tumor vaccine treatment and CAR-T cell immunotherapy, as novel strategies of adoptive cellular immunotherapy, have been developed rapidly in recent years. In particular, the achievements of CAR-T cell immunotherapy in hematological malignancies make the concept of immunooncology impressive [6, 7].

In the context of the numerous breakthroughs in immunooncology, more attention should be paid to the developing topics of research, such as intracellular antigens. The intracellular antigens, as the name implies, are generated intracellularly and then degraded by the proteasomes and presented in the context of the MHC-I signaling pathway as MHC/peptide complexes on the tumor surface. Intracellular antigens account for almost 95% of the tumor antigens [8–11]. The antibodies that have received FDA approvals and reached the market are almost all directed towards extracellular antigens, not intracellular ones. To target these antigens, a specific group of antibodies called T cell receptor- (TCR-) like/mimic antibodies has been developed in the preclinical process. They recognize the MHC/peptide complexes as well as the genuine TCRs and will hopefully broaden the spectrum of monoclonal antibodies applications and provide directions to future research [12–17].

**Figure 1:**
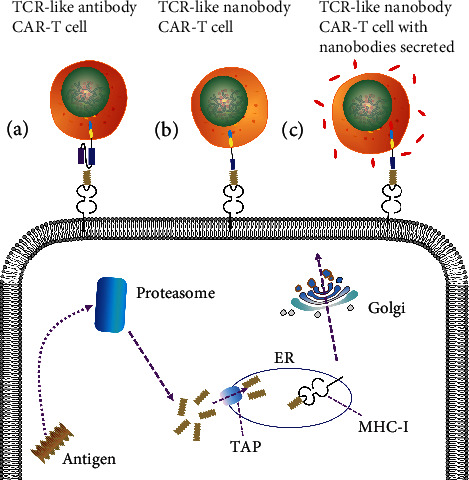
TCR-like CAR-T cell therapies for intracellular antigens degraded and presented on the tumor surface in the form of MHC/peptide complexes. Three of the possible methods are illustrated. (a) Conventional TCR-like antibody CAR-T cell therapies, with scFv to recognize the MHC/peptide complex and then trigger T cell activation and proliferation. (b) VHHs substituting conventional antibodies as the extracellular antigen-binding domains for the TCR-like nanobody CAR-T cell therapies. (c) VHHs simultaneously secreted in an intratumoral immune environment for the TCR-like nanobody CAR-T cell therapies, aside from the VHHs engineered into extracellular antigen-binding domains.

To improve the TCR-like antibodies with therapeutic potential, the TCR-like antibodies associated with CAR-T therapy were initially developed in 2001. They functioned to specifically recognize the MHC/peptide complexes and subsequently triggered T cell activation and proliferation under the potent costimulation signals [18, 19]. So far, the applications of CAR-T employed using TCR-like antibodies are uncommon (Table 1). The CAR designs depend on an extracellular antigen-binding domain, a hinge region, a transmembrane domain, and an intracellular domain that transmits the activation signals. These molecules are classified into three generations and varied in the quantity of costimulatory domains. As is well known, the first generation comprises CD3z only, the second generation adds one costimulatory domain such as 4-1BB (CD137), CD28, or OX40 (CD134) onto the backbone of the first generation, and more than one costimulatory domain was added in the third generation [20]. Any small modification in any moieties of the CAR construct may exert a significant influence. The same is true of the limited TCR-like antibody CAR-T therapies, varying from diverse antigen-binding modules derived from phage display or hybridoma to the various costimulatory domains, which may improve the therapeutic effect to some extent (Figure 1(a)) [21]. Therefore, there is much potential to be explored.

In this perspective article, we elaborate on the current status and limitations of the small number of TCR-like CAR-T therapies for intracellular antigens and imagine the alternative improvements in CAR-T engineering, with special emphasis on the single-domain antibodies (nanobodies), and provide discussion of possible initiatives in the future.

## 2. Current Situations and Limitations with TCR-Like CAR Targeting

TCR-like CAR-modified T cells show several advantages over conventional CAR-T expressing extracellular antigens. CAR-T therapy for intracellular antigens (also named as the TCR-like antibody CAR-T therapy) extends the therapeutic field to most of the unexploited tumor antigens, the intracellular antigens. They have been shown to account for 90% of tumor antigens [10]. In spite of that fact, the quantity of the therapeutics targeting intracellular antigens remains lower than the conventional CAR-T therapy for the extracellular antigens. The therapeutic efficacy has been verified due to the specificity of the TCR-like antibody and successful CAR-T cell activation and tumor lysis [18, 19, 22–27]. The TCR-like antibody CAR-T therapy circumvents the shortage of the specific biomarkers on the surface of tumor cells, achieving a few breakthroughs in solid tumors [28]. It has been reported that preserving the specificity of the TCR-like CAR to recognize the MHC/peptide complex is complicated; thus, any minimal binding to HLA may lose the CAR specificity owing to the high affinity of clustered CAR. Maus et al. have verified that the low-affinity DN-28z CAR, which is an attempt to maintain the specificity, was able to trigger T cell activation and cytotoxicity [25]. In general, besides the mutation in structure to maintain the specificity, TCR-like CAR-T shows superiority mainly in the broad target range to intracellular antigens and therapeutic potential for solid tumors.

At the same time, the reasons for the limitations of therapeutic efficacy in TCR-like CAR-T therapy might be multifaceted. The phage library-derived TCR-like antibody is converted into a CAR that is transduced into normal T cells, resulting in the construction of the TCR-like antibody CAR-T. The concerns and hurdles in improving the TCR-like antibody should be a focus when the therapy is being developed. The first concern is the coverage limitation of the targeted antigens, given the antibody is effective on certain MHC allele and certain peptides of tumor antigens, simultaneously and indispensably. Though HLA-A2 is the dominant allele in patients and, together with other HLA alleles, makes up the majority of the worldwide patients' MHC alleles, the specific peptides of tumor antigens that are relevant to the MHC alleles themselves remain insufficient [10, 29, 30]. Another important hurdle is the low productivity deriving from phage display or hybridoma techniques, which have been verified to be tough and, at the same time, time-consuming and costly [31–33]. In addition to all of this, the efficiency of tumor destruction mediated by the TCR-like antibody is not potent enough. An immune-suppressive environment could be one reason for that, and once the T cells gain access to the tumor cells, the tumor cells quickly hide themselves and secrete immune-suppressive cytokines that trigger the depletion or death of the natural T cells, monocytes, macrophages, or NK cells [34, 35]. The secreted cytokines include interleukin 10 (IL-10), which might promote T regulatory cell (Treg) suppressive activity or increase the expression of the immunosuppressive molecule like HLA-G. Meanwhile, the transforming growth factor beta (TGF-*β*), which has a well-known immunosuppressive role, garners much attention in this field [36–40].

In the end, tumor cells might evade immune surveillance because of absent or downregulated expression of MHC/peptide complexes on their surface. However, there is evidence suggesting that a few cytokines and chemicals can reverse the situation, activating the MHC signaling pathway and upregulating the MHC expression [41–43]. To some extent, the aforementioned problems could be the explanation for the rareness of CAR-T therapy for intracellular antigens, and further initiatives are urgently needed.

## 3. Probable Optimized Initiatives in Improving TCR-Like CAR-T Engineering

### 3.1. Single-Domain Antibody Targeted TCR-Like CARs

**Figure 2:**
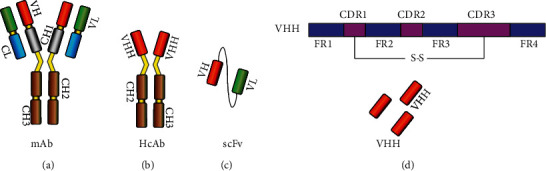
Formats of various antibodies. (a) Monoclonal antibody. (b) Camel heavy chain antibody. (c) Single-chain antibody fragment. (d) Single-domain antibody or nanobody derived from camel heavy chain antibody.

In order to promote the therapeutic efficacy of a TCR-like antibody CAR-T with higher antigen-binding specificity and productivity and, at the same time, to seek a substitute for the time-consuming and costly conventional antibody, researchers are focusing their attention on the single-domain antibody. The concept of the camelid single-domain antibody (sdAb) or heavy chain variable domain (VHH), as we now know, refers to the nanobody. The nanobodies that were initially reported were derived from the heavy chain antibodies (HCAbs), which were found in the dromedary (Arabian camel) serum samples and were analyzed by Hamers-Casterman and his colleagues [44]. HCAbs are composed of two heavy chains, each with a variable domain (VHH) and a constant region lacking CH1, which are linked with a disulfide bond to form homodimers [45]. To some extent, the structural features of the VHH show superiority as the smallest antigen-binding domain of HCAbs, which include the longer complementarity determining region 3 (CDR3) extending out to bind to the concave epitopes that a conventional antibody cannot bind. Furthermore, hydrophobic amino acids are involved in the paratope of the CDR1 and CDR2 and even more residues react with the antigen in the framework region (FR) (Figure 2) [46–48].

The primary superiority of nanobodies lies in its small size, having a molecular mass of 12-15 kDa, allowing for penetration of tumor-like compact tissues [49, 50]. The acquisition of nanobodies, usually described as the phage display technique, is associated with ease of production and low cost [51–54]. Furthermore, high affinity and specificity should not be ignored based on the structural features mentioned above, dealing with the covert epitopes that common antibodies cannot reach [55–57]. Other advantages of nanobodies lie in the low immunogenicity, good solubility, and high stability in the varied temperatures and pH [58]. As a result, nanobodies are involved in continual experimental and clinical trials, especially in the rising CAR-T therapy field [59–68]. However, the limited nanobody-based CAR-T engineering is mostly directed to extracellular antigens, such as tumor-associated markers like MUC1 and CD38, rarely to the intracellular antigens.

It has been reported that the TCR-like nanobody GPA7, specifically recognizing gp100 209–217/HLA-A2, was engineered into GPA7-28z CAR and mediated the effective cytotoxicity against human melanoma cells together with HLA-A2 following GPA7-28z CAR transduction into T cells [69]. The possibility of this hypothesis was demonstrated, even if it is rare (Figure 1(b)). To conquer the outgrowth of antigen escape variants, a tandem of two nanobodies specific for HER2 and CD20 constitutes the bispecific CAR, inducing T cell activation and tumor lysis [67]. At the same time, nanobody-based CARs have targeted the tumor stroma markers such as PD-L1 and VEGFR2, which have a direct effect on the tumor microenvironment and delay tumor growth [60, 62]. The studies mentioned above inform the TCR-like nanobody CAR-T therapies with a tandem of two TCR-like nanobodies specific to a certain type of cancer or an addition of a nanobody targeting a certain type of immune checkpoint inhibitor, which provides an option for the improvements that could be made in the nanobody-based antigen-binding domain in TCR-like nanobody CAR.

### 3.2. Probable Single-Domain Antibody Secreted TCR-Like CARs

The inefficiency of tumor destruction mediated by TCR-like antibody CAR-T might be due to the immune-suppressive environment and tumor immune escape, which might also hamper the related studies. For instance, the incomplete duplication of the tumor immunosuppressive microenvironment in in vitro and in vivo models could be a challenge in the success of a solid tumor study [70]. To conquer this, the armored CAR-T with genetic modifications added to the second- or third-generation CAR sequences leads to secretion of cytokines and proinflammatory ligands into the tumor microenvironment and improves efficacy and persistence of CAR-T. IL-12, CD40L, and 4-1BBL are often considered as possible targets to augment the potency of dendritic cells (DCs) and macrophages or restrain the Treg inhibition in the tumor microenvironment [71–76]. For example, Pegram et al. reported the enhanced tumor eradication in a syngeneic mouse model with armored anti-CD19-28*ζ*/IL-12 CAR-T cells. In the same study, it was demonstrated that the secreted IL-12 was involved in resistance to Tregs through IL-12 receptors on the CAR-T cells, which was in an autocrine mode [72]. It follows that moderate cytokine release might contribute to the improvement of the efficacy of TCR-like antibody CAR-T.

Apart from the cytokines and proinflammatory ligands secreted by CARs, single-domain antibodies (VHHs or nanobodies) can also be secreted by CAR-T, changing the intratumoral immune environment, thus contributing to good functions of CAR-T cells (Figure 1(c)). Yushu et al. demonstrated that the anti-CD47 VHH secreted by CAR-T stimulated the macrophage engulfment systematically and that anti-CD47 VHH-Fc fusions secreted locally enhanced safety compared to the systemic applications. At the same time, the secreted anti-PD-L1 or anti-CTL-4 VHH contributed to the persistence of CAR-T cells [77].

As the secretion of VHHs by CAR-T has been proved to be probable, this suggests the possibility of TCR-like CAR-T secreting VHHs that could specifically recognize the MHC/peptide complexes, weaken the impact of the tumor immunosuppressive microenvironment, and reinforce the innate immune system.

### 3.3. Selection of Tumor Antigens

To exploit the targeted intracellular antigens for TCR-like CAR-T therapies, researchers should focus on the promising antigens with the features listed below, which might provide potential targets for TCR-like CAR-T therapies.

Intracellular antigens account for more than 90% of tumor antigens which are classified into three groups according to various features: oncovirus antigens, neoantigens or tumor-specific antigens, and cancer-testis antigens or tumor-associated self-antigens [17]. The oncovirus antigens like latent membrane protein 1 (LMP1), LMP2 and Epstein-Barr nuclear antigen 1-3 (EBNA 1-3), originating from Epstein-Barr virus (EBV), and hepatitis B virus X protein (HBX), derived from hepatitis virus (HBV), are localized to the cytoplasm, presented on the cell surface through the antigen presentation pathway, and are specialized in the oncoviruses with high tumor specificity [78–80]. Neoantigens or tumor-specific antigens are generated when gene/chromosome mutations occur in tumor cells [81–83]. The mutations, like the chromosomal translocation, duplication, or loss in every region of the genes, lead to neoantigens such as the alpha-actinin-4 K122N in lung cancer and beta-catenin S37F in melanoma [84–86]. Cancer-testis antigens or tumor-associated self-antigens such as melanoma antigen-1 (MAGE-1), WT1, NY-ESO-1, and synovial sarcoma X (SSX) are not only expressed in tumor cells but also partly in normal tissues such as embryonic or germ cells [87, 88]. Among all the potential targeted antigens, cancer-testis antigens or tumor-associated self-antigens stand out in the existing experimental results [13, 89].

With the potential targeted antigens identified, it could be possible for them to be processed and presented in the form of peptides on the cell surface. From using T cell epitope cloning to identify human tumor antigen MAGE-1 in the 1990s for the first time to exploiting serological analysis of recombinant cDNA expression libraries (SEREX) to identify antigens with high immunogenicity like NY-ESO-1, scientists have focused mainly on the immunological methods [90–92]. However, there is still a long way to go in the identification of the tumor antigens. Since then, other techniques such as chromatography and mass spectrometry, DNA microarray, and reverse immunology have been developed, and mass spectrometry shows superiority in identifying the tumor antigen peptides in a direct way [93].

### 3.4. Other Possible and Novel Approaches for CAR

To synchronously limit antigen escape and minimize toxicities, the switchable universal CAR comprising the target module (TM) of tumor-specific antibody and a UniCAR-T without specificity to the tumor but to the short peptide tagged on the TM has been introduced. It is the TM that provides the UniCAR with specificity, and once the tumor is not around, the UniCAR remains inactive. In the application of UniCAR to the bivalent epidermal growth factor receptor (*α*-EGFR-EGFR), the TM that retargeted UniCAR-T to the tumor cells with low-expressing EGFR has proved to be effective [94, 95]. A split, universal, and programmable (SUPRA) CAR system engineered as a potent tool against genetically unsteady or highly heterogeneous tumor types, comprises two parts: (1) a zipCAR that is almost identical to the conventional structure of CAR but with a leucine zipper in the extracellular domain and (2) a zipFv added externally with the ligand-binding domain and fused to a second leucine zipper on the zipCAR, playing a significant role in antigen-binding specificity [96, 97]. As a result, the efficiency of zipCAR could be varied due to the quantity and quality of input zipFvs, which broaden the spectrum of remediable malignancies. It has been observed that reducing the zipFv input or binding affinity of zipFv and zipCAR through the leucine zipper would decrease the interferon-*γ* (IFN-*γ*) production and tumor lysis [98–100].

The extraordinary breakthroughs in bioengineering offer new thoughts to the progress of CAR-T, expanding the probable therapeutic possibilities for intracellular antigens.

## 4. Conclusion

The limited TCR-like antibody-based CAR-T cell therapies have not entered into clinical trials, and we highlight here a number of possible reasons for this. However, once these issues are resolved, the progress of CAR-T targeting intracellular antigens can be potentially improved. In particular, the high yields and ease of production of nanobodies might settle the production difficulties, and nanobodies engineered or secreted in CAR-T might augment the specificity to recognize the MHC/peptide complexes and improve the immune-suppressive environment. The clinical trial of CAR-T engineering with a CD19/CD20 bispecific nanobody was newly introduced, and whether a safer and preferable CAR-T could be brought still needs to be awaited, let alone the intracellular antigen-targeted TCR-like nanobody CAR-T therapies that remain in the bud [101]. A phase *Ι* study demonstrated that immunogenic responses could still occur when the TAS266 (a DR5 receptor-targeted agonistic tetravalent nanobody) is administrated [102]. Simultaneously, we are waiting for more intracellular antigens to be identified and other outstanding advances in biological engineering and synthetic biology as new strategies to apply to the CAR-T therapies for intracellular antigens.

## Figures and Tables

**Table 1:** TCR-like antibody CAR-T cell therapy in human diseases.

Antigen	Epitope sequence	MHC allele	Clone	Generation strategy	CAR sequence	Human disease	Reference
MAGE-1	EADPTGHSY	HLA-A∗0101	Fab-G8	Phage	Fab-G8-CD4/*γ*	Melanoma	[18]
	EADPTGHSY	HLA-A∗0101	Fab-G8/Fab-Hyb3	Phage	Fab-G8/*γ* Fab-Hyb3/*γ*	Melanoma	[22]
Proteinase 3	VLQELNVTV	HLA-A∗0201	8F4	Hybridoma	8F4-28Z	AML	[24]
WT1	RMFPNAPYL	HLA-A∗0201	F2, F3	Phage	F2-28Z/F3-28Z	Leukemia	[26]
	RMFPNAPYL	HLA-A∗0201	Clone 45	Phage	Clone45-4-1BBZ	Leukemia	[23]
HA-1H	VLHHDLLEA	HLA-A∗0201	#131	Phage	#131-28Z	Leukemia	[19]
NY-ESO-1	SLIMWITQC	HLA-A∗0201	T1	Phage	T1-28Z	Melanoma	[25]
AFP	FMNKFIYET	HLA-A∗0201	ET1402L1	Phage	ET1402L1-28Z	Liver cancer	[27]

Notes: published TCR-like antibody CAR-T targeting cancer antigens are summarized. MHC: major histocompatibility complex; CAR: chimeric antigen receptor.
